# Idiopathic basal ganglia calcification presenting with obsessive‐compulsive symptoms: A case report

**DOI:** 10.1002/pcn5.166

**Published:** 2024-01-18

**Authors:** Daisuke Yoshioka, Takehiko Yamanashi, Kenta Taneda, Takashi Matsukawa, Kenta Orimo, Masaaki Iwata

**Affiliations:** ^1^ Division of Neuropsychiatry, Faculty of Medicine Tottori University Yonago Japan; ^2^ Division of Neurology, Faculty of Medicine Tottori University Yonago Japan; ^3^ Department of Neurology, Graduate School of Medicine The University of Tokyo Tokyo Japan

**Keywords:** Fahr's disease, idiopathic basal ganglia calcification, obsessive‐compulsive symptoms

## Abstract

**Background:**

Idiopathic basal ganglia calcification (IBGC), also known as Farh's disease, is a rare neurodegenerative disorder characterized by calcification of the basal ganglia and other brain regions. This disease usually occurs in middle‐aged patients and presents with various neurological and psychiatric symptoms. The exact prevalence is unknown; however, population genomic data analysis suggests a prevalence of at least 4.5/10,000 to 3.3/1000, indicating that the disease is more common than previously thought and remains underdiagnosed.

**Case Presentation:**

We report the case of a middle‐aged Japanese man who attempted suicide twice because of obsessive‐compulsive ideation caused by trivial triggers. The patient's psychiatric symptoms resolved relatively quickly after hospitalization, and imaging and genetic testing led to a diagnosis of IBGC.

**Conclusion:**

This case report illustrates the importance of including IBGC in the differential diagnosis of psychiatric symptoms that initially develop in middle‐aged patients.

## Graphical abstract

Idiopathic basal ganglia calcification (IBGC) is a rare neurodegenerative disorder characterized by calcification of the basal ganglia and other brain regions. A middle‐aged Japanese man who attempted suicide twice because of obsessive thoughts was finally diagnosed with IBGC by imaging and genetic testing. This case report illustrates the importance of including IBGC in the differential diagnosis of psychiatric symptoms that initially develop in middle‐aged patients.

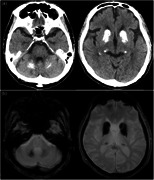

## BACKGROUND

Idiopathic basal ganglia calcification (IBGC) (Farh's disease) is a rare neurodegenerative disorder characterized by the calcification of the basal ganglia and other brain regions. Both familial and nonfamilial cases have been reported, predominantly with an autosomal dominant fashion.^1^, ^2^ The exact prevalence is unknown; however, the minimal IBGC prevalence from population genomic data analysis is estimated to range from 4.5/10,000 to 3.3/1000, using different evidence levels for pathogenicity,^3^ suggesting that IBGC is more common than previously thought and remains underdiagnosed. IBGC is a progressive disease; abnormal calcium deposition generally begins in the third decade of life and neurological symptoms appear two decades later.^4^ However, some cases can occur during childhood or later in life.^5^ Movement disorders are the most common IBGC symptoms, accounting for 55% of the total symptomatic patient population. Parkinsonism was observed in 57%, chorea in 19%, tremors in 8%, dystonia in 8%, athetosis in 5%, and facial dyskinesia in 3% of cases.^6^ Psychiatric features are the primary symptoms in approximately 40% of the patients, and the common psychiatric features are mood disorders, cognitive deterioration, and psychotic symptoms.^7^ The pathogenesis of the psychiatric manifestations of IBGC is not well known, and the psychiatric symptoms are diverse and vary in severity from case to case. We report a case of IBGC in a middle‐aged Japanese man who experienced obsessive thoughts triggered by trivial events, with repeated suicide attempts.

## CASE PRESENTATION

A 55‐year‐old Japanese man presented with excessive anxiety about kidney cancer recurrence. The patient was married with two sons—the first son with severe mental retardation and the second son with attention deficit hyperactivity disorder. However, the patient's own history contained no evidence suggesting an intellectual disability.

**Figure 1:**
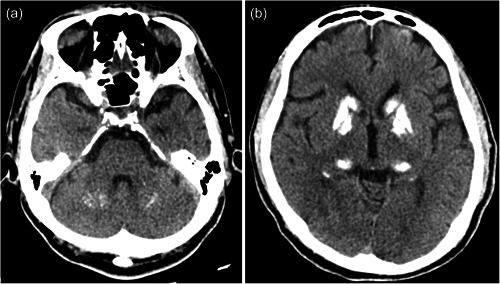
Evidence of bilateral calcification of the basal ganglia, thalamus, and dentate nucleus on brain computed tomography scan.

The patient underwent surgery for cancer of the left kidney at 47 years of age, and the postoperative course was uneventful. At 55 years of age, a health check‐up revealed hematuria, which increased his anxiety about recurrence. One month after the checkup, the patient gradually became less energetic. Although a detailed examination revealed no recurrence, his anxiety persisted. Three months after the check‐up, the patient was diagnosed with depression in a psychiatric clinic and was started on pharmacological treatment. However, owing to his mental illness, the patient was obsessed with the fear that he might be dismissed from his job. The next day, the patient attempted suicide by hanging. The patient's younger son foiled the attempt and brought him to our hospital. The patient did not respond to calls on arrival at our hospital but resisted medical procedures with his eyes closed, and a dissociative stupor was suspected. The patient soon awoke and was able to speak, and a psychiatric evaluation was performed. Although the patient described vague anxiety, a depressive mood was not evident even after this serious suicide attempt. No significant physical abnormalities were observed, and the patient was admitted to the psychiatric department for examination and treatment of psychiatric symptoms. Bilateral calcifications of the basal ganglia, thalamus, and dentate nucleus were noted for the first time on head computed tomography (CT) (Figure 1). To manage the patient's severe anxiety and confusion, antidepressant and antipsychotic treatment was initiated, and his mental condition improved. Finally, he was diagnosed with depression, and 2 months after admission, he was discharged from the hospital and returned to work. Olanzapine (2.5 mg), sulpiride (100 mg), aripiprazole (3 mg), and valproic acid (200 mg) administration was continued for treating depression; however, the antipsychotics caused drug‐induced parkinsonism, with symptoms such as tremors and brachybasia. Additionally, the patient often complained of other physical anxieties and continued to experience emotional instability.

At the age of 63, a change in the name of the company he worked for triggered new anxiety about his future, believing that he would be laid off. Additionally, the patient gradually became more confused and obsessive, and wondered if his smartphone used for work had been infected with a virus or if he might be infected by the coronavirus. Although the patient purchased a new smartphone, he could not sleep at night because he panicked that the screen would change on its own. The patient subsequently wrote a suicide note to his wife and attempted suicide by overdosing on sleeping pills. The family found him lying unconscious in bed and admitted him to our hospital. The patient unemotionally informed us that he had taken an overdose of pills because of confusion and denied the current presence of suicidal ideation. As in the previous admission, the depressive mood was not apparent; however, the patient requested hospitalization for rest and was admitted to the psychiatric department. Before admission, the patient had been taking aripiprazole (0.75 mg), valproic acid (200 mg), biperiden (2.5 mg), flunitrazepam (2 mg), and brotizolam (0.25 mg). All medications were temporarily discontinued after this episode of overdosing, and the patient was followed up.

**Figure 2:**
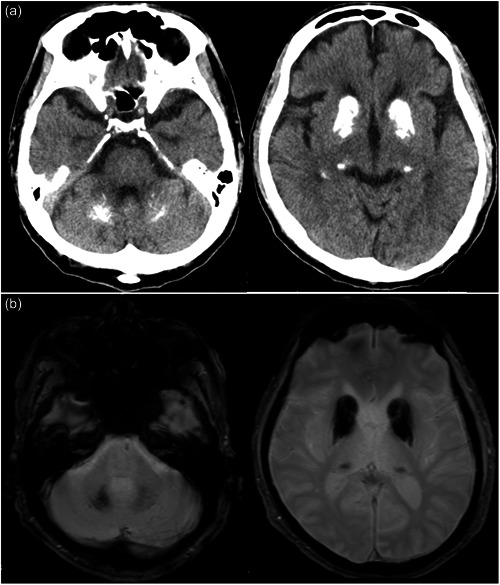
(a) The brain computed tomography (CT) scan revealed symmetrical calcification in the basal ganglia, thalamus, and dentate nucleus, with the extent of calcification slightly larger than before. (b) T2‐star‐weighted magnetic resonance imaging showed calcification in the same areas as did the CT scan.

**Figure 3:**
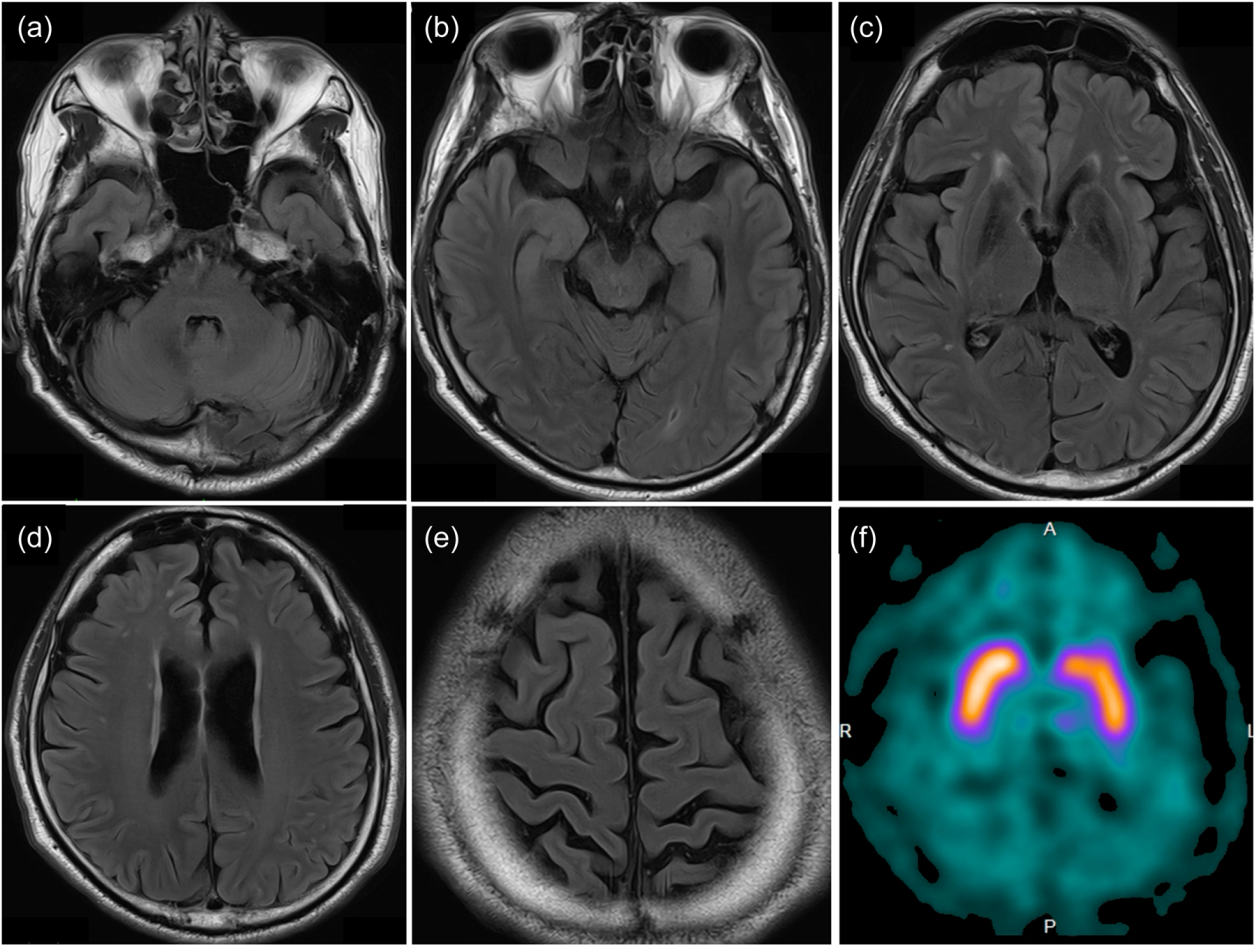
Fluid‐attenuated inversion recovery sequence brain magnetic resonance imaging showed no abnormal findings other than calcified lesions. Axial brain slices at the level of (a) pons, (b) hippocampus, (c) basal ganglia, (d) central part of lateral ventricle, and (e) central sulcus. (f) Dopamine transporter scan showed decreased in the bilateral putaminal specific binding ratios (4.43 and 4.22 for the right and left side, respectively).

Neurological examination findings on admission were normal, except for a slight bilateral tremor in the hands and a masked face. On mental status examination, the patient was oriented and cooperative; however, he spoke without much emotional expression. A brain CT scan revealed symmetrical calcification in the basal ganglia, thalamus, and dentate nucleus, with the extent of calcification slightly larger than before (Figure 2a). After hospitalization, the patient did not exhibit any notable psychiatric symptoms; however, on the ninth day of hospitalization, he suddenly became agitated and began screaming that he had been deceived and admitted to the hospital. The next day, however, the patient fell into a stupor with his eyes closed. Eventually, these symptoms spontaneously disappeared, and the patient apologized for having been distraught. Subsequent tests indicated no cognitive dysfunction (Mini‐Mental State Examination score 29/30). To thoroughly examine the patient's psychiatric symptoms and parkinsonism, magnetic resonance imaging (MRI) of the head and dopamine transporter (DAT) imaging were performed. MRI showed calcification in the same areas as did the CT scan; however, no other abnormal findings were observed (Figures 2b and 3a–e). DAT scans showed decreased bilateral putaminal specific binding ratios (4.43 and 4.22 for the right and left sides, respectively) (Figure 3f). Laboratory values of parathyroid hormone, thyroid‐stimulating hormone, T3, and T4 were within normal reference limits. Therefore, the possibility of secondary calcification caused by metabolic or endocrinological disorders was excluded. Genetic analysis revealed a heterozygous *SLC20A2* nonsense mutation (c.514A>T, p.Lys172Ter in exon 4), whose pathogenesis has been previously reported, confirming the clinical diagnosis of IBGC. The previously recognized obsessive‐compulsive symptoms and anxiety were considered concomitant symptoms of IBGC. The patient's psychiatric symptoms spontaneously improved, and valproic acid (200 mg) was resumed to prevent mood instability. No adverse effects, such as worsening of parkinsonism, were observed, and the patient was discharged from the hospital within a month. A head CT scan performed on the 28‐year‐old second son revealed calcification of the basal ganglia, confirming that the disease was familial in origin.

## DISCUSSION

IBGC is characterized by calcification of the bilateral basal ganglia. It usually occurs in middle‐aged patients, who present with various neurological and psychiatric symptoms. CT is useful for detecting calcifications. IBGC diagnosis requires the exclusion of metabolic diseases, especially those related to calcium and phosphorus, autoimmune diseases, infections, and poisoning, which can lead to calcification in the brain. Contrastingly, age‐related and asymptomatic physiological basal ganglia calcifications have been detected in up to 20% of cases on routine CT scans. However, radiologic patterns may distinguish it from pathologic calcification, which generally involves the globus pallidus and is bilateral and pale.^8^ Additionally, DAT scanning is useful for detecting dopamine dysfunction in the basal ganglia and can help differentiate parkinsonism. In the present case, imaging revealed bilateral decreases in presynaptic dopaminergic function. Inconsistent DAT scan results have been reported in patients with IBGC; however, in most cases, a decreased presynaptic dopaminergic function was observed.^9^, ^10^, ^11^, ^12^ Genetic testing should be considered if secondary causes have been ruled out and the family history suggests autosomal dominant inheritance.^13^ *SLC20A2* is the most common gene involved in IBGC, followed by *PDGFB*, *PDGFRB*, and *XPR1*. *SLC20A2* encodes type III sodium phosphate transporter 2, whose loss‐of‐function mutation may lead to the local accumulation of inorganic phosphate in the brain, leading to calcium phosphate deposition.^14^

After excluding secondary causes in the present case, genetic testing confirmed the presence of the *SLC20A2* nonsense mutation (c.514A>T, p.Lys172Ter in exon 4), which has been reported to be pathogenic.^15^ Additionally, calcified lesions in the brain were detected in the younger son, confirming IBGC diagnosis.

The clinical manifestations of this case were uncorrectable obsessions triggered by trivial events, leading to suicide attempts and parkinsonism as motor symptoms. The clinical manifestations of IBGC vary and have not been consistently reported. However, mood disorders were present in more than half of cases of IBGC with psychiatric symptoms, and the other psychiatric symptoms included cognitive dysfunction and psychotic symptoms.^7^, ^16^ In contrast, several cases of IBGC with obsessive‐compulsive symptoms have been reported.^17^, ^18^, ^19^ Our patient had various symptoms, including anxiety, hopelessness, dissociative symptoms, and suicide attempts, all of which were triggered by obsessive‐compulsive ideation. Therefore, we considered the obsessive‐compulsive symptoms to reflect the patient's primary pathophysiology and the other symptoms to be concomitant. The underlying mechanisms for psychiatric symptoms in IBGC are not precisely known but are thought to include the effects of impaired basal ganglia function due to calcification, resulting in negative effects on the frontal cortex, which is largely associated with the basal ganglia,^20^, ^21^, ^22^ and other factors, such as abnormal calcium metabolism in the brain.^6^ The basal ganglia have been suggested to interact with the prefrontal cortex and thalamus via the fronto‐striato‐thalamic circuit to provide cognitive flexibility.^23^ Notably, a lack of cognitive flexibility has been reported in patients with Parkinson's disease and stroke with focal damage to the basal ganglia.^24^, ^25^, ^26^ The association between cognitive inflexibility and obsessive‐compulsive disorder (OCD) has also been reported,^27^ where Sternheim et al. stated that negative thoughts relevant to OCD, such as worry and obsessions, may simply fill up the cognitive space available for patients, resulting in fewer available cognitive tools to get through situations that require flexibility. Furthermore, low DAT levels in the basal ganglia of patients with severe hopelessness and severe suicidality and a positive correlation between severe hopelessness and dissociative symptoms have been reported.^28^ This suggests that dopaminergic function in the basal ganglia may be involved in hopelessness and the control of suicidal tendencies. In the present case, hematuria noted during the medical examination and a change in the name of the company in which he was employed triggered obsessive thoughts, which led to anxiety, dissociative symptoms, and two suicide attempts. These manifestations may be related to calcifications in the basal ganglia and consequent cognitive inflexibility and dopaminergic system abnormalities.

Specific treatments to control calcification progression in the basal ganglia in IBGC are not available, except for a theoretically unconfirmed report on the use of chelators with antioxidants and calcium antagonists,^29^ and therefore only symptomatic treatment is provided to improve neuropsychiatric symptoms. Patients treated with IBGC are extremely sensitive to extrapyramidal side effects,^30^ which were also noted in the patient in this case study. During treatment, these side effects should be carefully monitored. Furthermore, electroconvulsive therapy has been reported to be effective in some cases.^31^

## CONCLUSION

The present case report emphasizes the importance of brain imaging and comprehensive clinical examinations when evaluating patients presenting with psychiatric and motor symptoms. IBGC should be included in the differential diagnosis, particularly in cases of calcified lesions in the brain.

## AUTHOR CONTRIBUTIONS

Daisuke Yoshioka treated the patient and drafted the manuscript. Kenta Taneda performed a neurological examination. Takashi Matsukawa and Kenta Orimo conducted genetic testing. Takehiko Yamanashi and Masaaki Iwata critically reviewed the draft and revised it. All authors approved the final version of the manuscript.

## CONFLICT OF INTEREST STATEMENT

The authors declare no conflict of interest.

## ETHICS APPROVAL STATEMENT

This study was conducted according to the principles of the Declaration of Helsinki.

## PATIENT CONSENT STATEMENT

Informed written consent and a signed release were obtained from the patient for the publication of this report and any accompanying images.

## CLINICAL TRIAL REGISTRATION

N/A.

## Data Availability

Data sharing is not applicable to this article.
